# Supplementary material for: Transcriptomic Classification of Pituitary Neuroendocrine Tumors Causing Acromegaly
Source: Cells. 2022 Nov 30;11(23):3846. doi: 10.3390/cells11233846 (PMC9738119; doi:10.3390/cells11233846)
Supplement: Supplementary file 1 [file cells-11-03846-s001.zip › Figure S1.pdf]

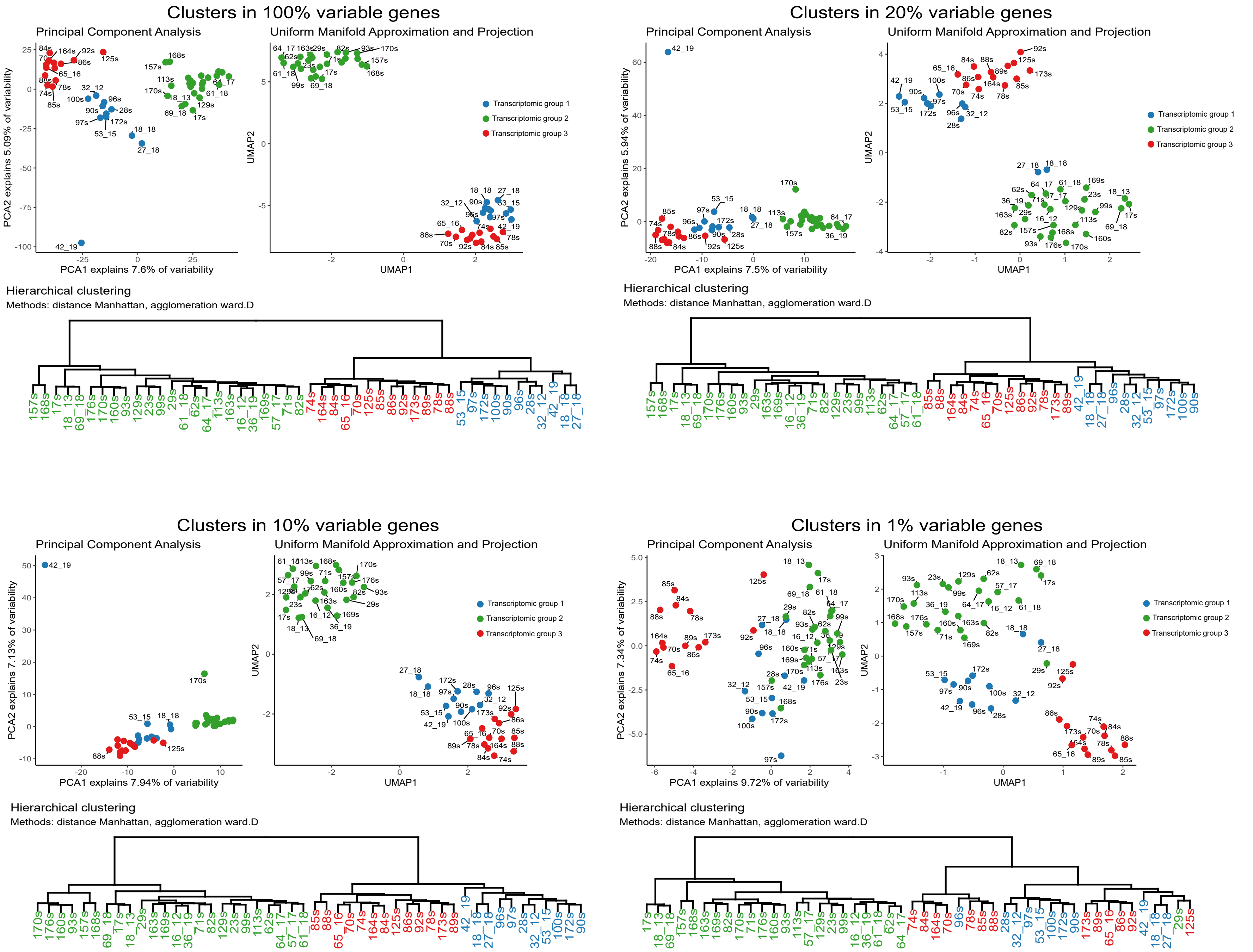

Figure S1. Overall gene expression profile in somatotroph tumors including Principal component analysis (PCA) and uniform manifold approximation and projection (UMAP) results and Hierarchical clustering based on the 100%, 20%, 10% and 1% of genes with most variable expression.
