# Supplementary material for: Transcriptomic Classification of Pituitary Neuroendocrine Tumors Causing Acromegaly
Source: Cells. 2022 Nov 30;11(23):3846. doi: 10.3390/cells11233846 (PMC9738119; doi:10.3390/cells11233846)
Supplement: Supplementary file 1 [file cells-11-03846-s001.zip › Figure S2.pdf]

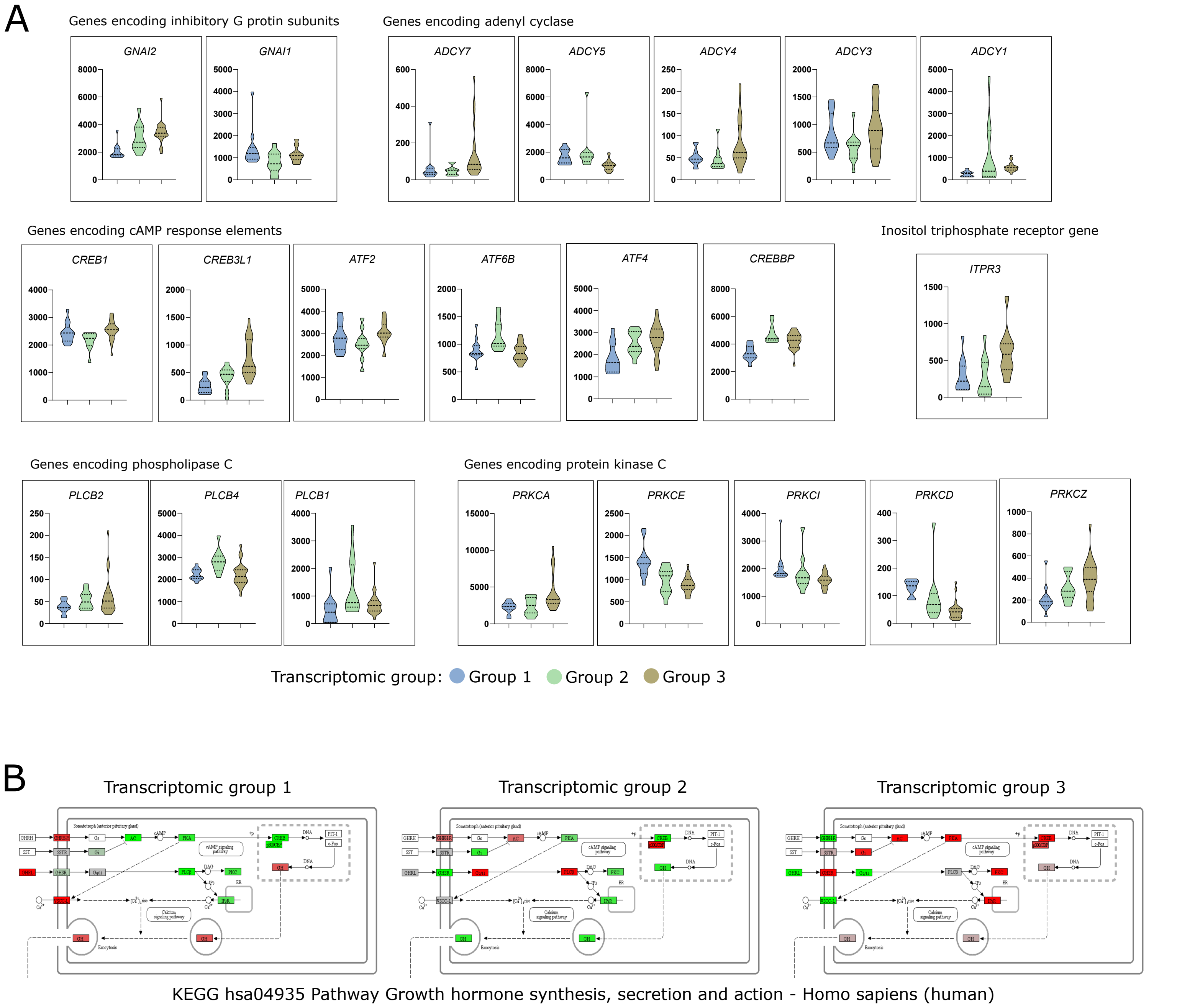

Figure S2. Difference in the expression of genes involved in growth hormone (GH) secretion pathway in three subtypes of somatotroph tumors. A. Expression levels of differentially expressed genes encoding inhibitory G-protein subunits, adenyl cyclases, cAMP response elements, phospholipases C, protein kinases C and inositol trisphosphate receptor; B Scaled normalized RNAseq read counts of genes encoding each element of GH synthesis pathway were visualized on KEGG pathway with pathview (original, unprocessed picture).
