# Supplementary material for: Transcriptomic Classification of Pituitary Neuroendocrine Tumors Causing Acromegaly
Source: Cells. 2022 Nov 30;11(23):3846. doi: 10.3390/cells11233846 (PMC9738119; doi:10.3390/cells11233846)
Supplement: Supplementary file 1 [file cells-11-03846-s001.zip › Figure S3.pdf]

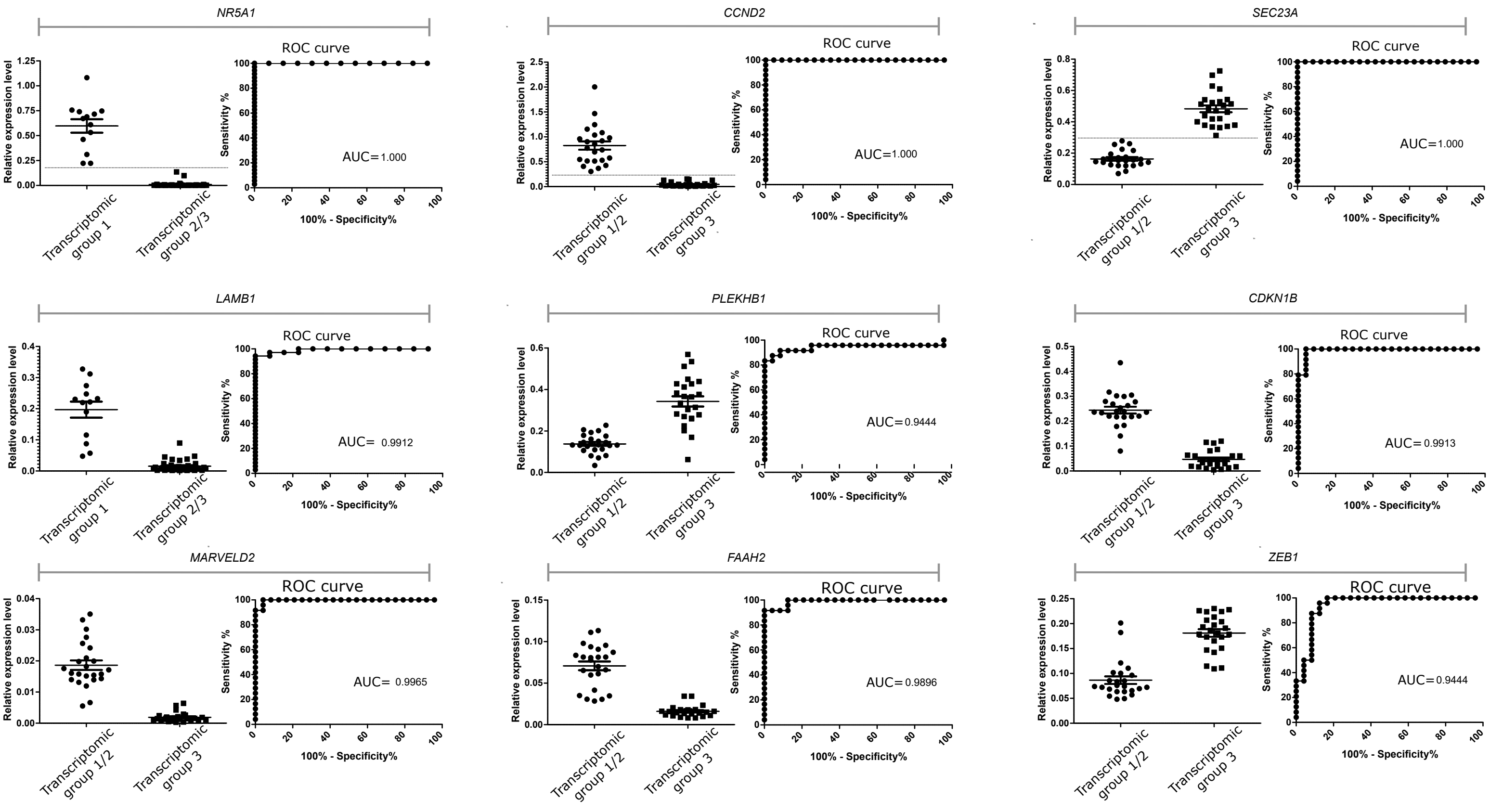

Classifier	Selected Marker gene	AUC based on RNAseq data	AUC based on qRT-PCR data
group 1 vs 2/3	*NR5A1*	1	1
groups 1/2 vs 3	*CCND2*	0,988	1
groups 1/2 vs 3	*SEC23A*	0,995	1
group 1 vs 2/3	*LAMB1*	0,996	0,991
groups 1/2 vs 3	*PLEKHB2*	1	0,944
groups 1/2 vs 3	*CDKN1B*	0,995	0,991
groups 1/2 vs 3	*MARVELD2*	1	0,996
groups 1/2 vs 3	*FAAH2*	1	0,989
groups 1/2 vs 3	*ZEB1*	0,997	0,944

Figure S3. qRT-PCR-based evaluation of 9 genes that potentially serve as marker genes for trasncriptional groups of somatotroph tumors. Candidate markers were selected using RNA-seq data; A. Scatter plot and ROC curve for each gene evaluation; dashed horizontal line indicate threshold value, ROC - receiver operating characteristicAUC - area uner curve; B. Summary of the evaluation of each marker candidate.
